# Phononic Band Gaps in 2D Quadratic and 3D Cubic Cellular Structures

**DOI:** 10.3390/ma8125463

**Published:** 2015-12-02

**Authors:** Franziska Warmuth, Carolin Körner

**Affiliations:** 1 Institute of Advanced Materials and Processes (ZMP), University of Erlangen-Nürnberg, Dr.-Mack-Str. 81, Fürth 90762, Germany; 2 Chair of Metals Science and Technology (WTM), University of Erlangen-Nürnberg, Martensstr. 5, Erlangen 91058, Germany; carolin.koerner@ww.uni-erlangen.de

**Keywords:** cellular materials, metamaterials, phononic band gaps, finite element method, dispersion relations

## Abstract

The static and dynamic mechanical behaviour of cellular materials can be designed by the architecture of the underlying unit cell. In this paper, the phononic band structure of 2D and 3D cellular structures is investigated. It is shown how the geometry of the unit cell influences the band structure and eventually leads to full band gaps. The mechanism leading to full band gaps is elucidated. Based on this knowledge, a 3D cellular structure with a broad full band gap is identified. Furthermore, the dependence of the width of the gap on the geometry parameters of the unit cell is presented.

## 1. Introduction

Cellular materials offer a variety of interesting properties that can hardly or not at all be achieved with compact materials. They are not only extremely suited for lightweight applications [1], but, at the same time, may exhibit completely new properties such as a negative Poisson’s ratio [2,3] or tunable thermal expansion coefficient [4]. Undesired vibrations or noises can be controlled with the help of wave absorbing or waveguiding materials [5,6]. Such materials are usually referred to as metamaterials. Their behaviour is governed by the properties of their unit cell. Tailoring the architecture of the unit cell, the properties of the cellular material can be designed. Due to the high geometric freedom of new manufacturing methods such as Selective Electron Beam Melting (SEBM) or Selective Laser Melting (SLM), these complex cellular materials can now be built and experimentally analyzed [7,8].

Phononic band gaps are frequency intervals in which no wave motion can occur. Therefore, they are also referred to as stop bands. In between those stop bands, so-called pass bands occur, where the wave can propagate through the lattice. The emergence of band gaps is extremely sensitive to the architecture of the unit cell.

A variety of simple unit cells was studied in [9] and band gaps were obtained for triangular and hexagonal honeycombs depending on their slenderness ratio. Ruzzene, *et al.* found only partial band gaps for the regular honeycomb structure. With the help of an optimization procedure, they identified an angle of $-30^{\circ }$ for the inverted honeycomb as optimal for deep and broad band gaps [10]. Bertoldi and Boyce used large deformations to induce shape deformations in a cellular structure to control its band gap [11]. It was also shown that the eigenmodes around the band gaps can be attributed to the eigenmodes of a single strut and mode transition was found to be the reason for the emergence of band gaps [12].

In this paper, the band gap behaviour of cellular structures based on the quadratic unit cell is studied with the help of dispersion relations. Eigenmode analysis is used as a systematic way for finding new structures from basic unit cells leading to bent struts and chiral nodal points. Cellular structures in 2D and 3D showing a full band gap are identified. The reason for the occurrence of band gaps is described and used to deduce a design principle for the creation of new two- and three-dimensional structures with phononic band gaps.

## 2. Methods

### 2.1. Dispersion Relations

**Figure 1:**
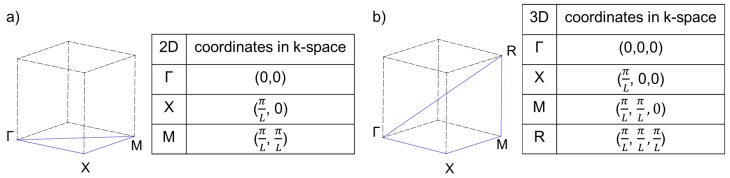
Two- (**a**); and three-dimensional (**b**) irreducible 1st Brillouin zone and corresponding coordinates of the path endpoints in $\vec{k}$-space.

To study the phononic behaviour of cellular structures, principles and methods from solid state physics can be applied [13]. It is convenient to study wave propagation in the wavevector space by defining a reciprocal lattice. Both the lattice and the reciprocal lattice consist of periodically arranged unit cells. According to Bloch’s principle, the wave propagation in a periodic lattice is governed by the wave propagation in a unit cell if the unit cell can be periodically arranged to form the lattice. The wave propagation is not dependent on its specific place in the periodic lattice. A plane wave description for the displacement $\vec{u}\left(\vec{r_{i}}\right)$ at a lattice point $\vec{r_{i}}$ would be:

(1) $$\vec{u}\left(\vec{r_{i}}\right)\;=\;\vec{u_{i}}\;e^{\left(i\;\omega \;t\;-\;\vec{k}\;\cdot \;\vec{r_{i}}\right)}$$

with $\vec{u_{i}}$ the amplitude, $\vec{k}$ the wave wector and *ω* the frequency of the plane wave. The wave motion can then be studied at the boundaries of the first Brillouin zone (see Figure 1) which corresponds to the Wigner-Seitz cell of the reciprocal lattice. An eigenvalue problem is defined

(2) $$\tilde{D}\;\left(k_{1}\;,\;k_{2}\;,\;k_{3}\;,\;\omega \right)\;\tilde{u}\;=\;0$$

with four variables $k_{1}$, $k_{2}$, $k_{3}$ and *ω* that describe a wave propagating at a frequency *ω* in the direction of the wave vector $\vec{k}$ consisting of $k_{1}$, $k_{2}$ and $k_{3}$. $\tilde{D}$ is the stiffness matrix. Solving this equation for the relevant directions $\vec{k_{i}}$ leads to the dispersion relations which describe the dependancy between a wave with a certain direction (described by the wave vector $\vec{k}$) and frequency *ω* of the excited lattice.

Only wave vectors that lead to frequencies that correspond to natural resonances of the lattice can pass through. If certain frequencies cannot excite the lattice to natural resonances, independent of the value of the wave vector, a so-called phononic band gap develops. These waves cannot propagate through the lattice. Partial band gaps are possible if wave propagation is possible in some directions and forbidden in others. A more detailed description can be found in [14].

### 2.2. Used Geometries

**Figure 2:**
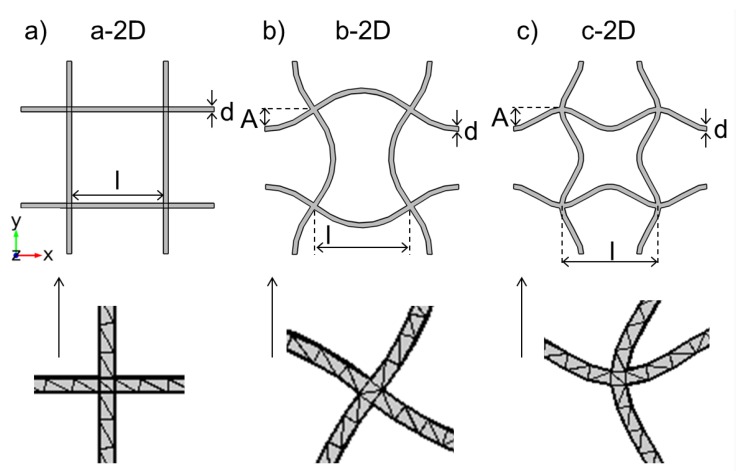
Two-dimensional unit cells and FEM mesh: (**a**) Basic cell; (**b**) Eigenmode with chiral nodes; (**c**) Eigenmode with fixed nodes.

With the help of the finite element method (FEM) software Comsol Multiphysics 5.0 dispersion relations of quadratic unit cells are studied. The chosen unit cells are depicted in Figure 2. Besides the basic structure a-2D, the simple quadratic unit cell with strut thickness d and nodal distance l, eigenmodes of the basic quadratic unit cell are also used. Calculating the eigenmodes of a basic unit cell displays all possible variations of this basic structure without changing the fundamental form of the used unit cell. Periodic boundary conditions are applied to guarantee that the obtained eigenmode shapes can still be arranged periodically.

Structure b-2D can be described as the eigenmode with chiral nodes as the nodes do not feature a rotative reflection axis (see Figure 2). The sines’ turning points meet in the nodal points. A is the distance of the arc extremum in the middle of the strut to the undeflected strut. In structure c-2D, the sines intersect at the nodal points (see Figure 2). When oscillating, the nodal points of this eigenmode stay at their initial position without rotation or other movements. Structure c-2D is named the eigenmode with fixed nodes. When arranged, the unit cells form periodic lattices.

**Figure 3:**
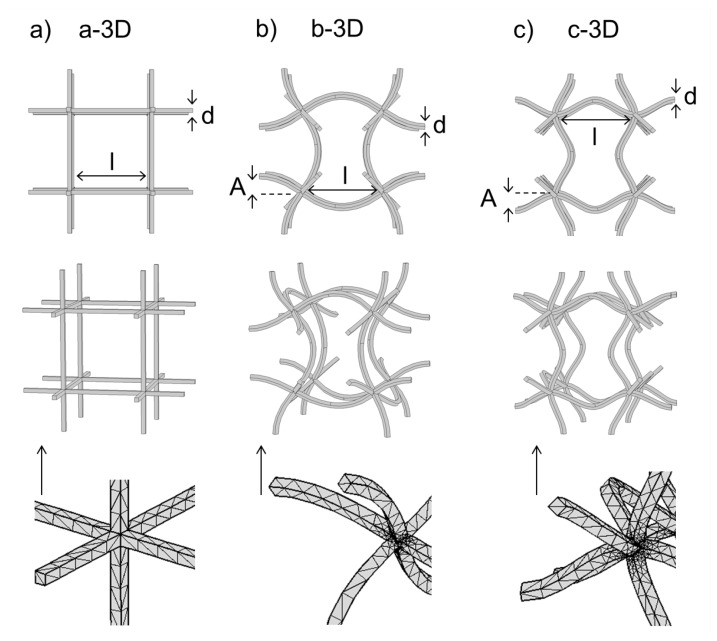
Three-dimensional unit cells: Upper row: xy-direction, middle row: scenography, lower row: mesh: (**a**) Quadratic unit cell; (**b**) Eigenmode with chiral nodes; (**c**) Eigenmode with fixed nodes.

The same construction principle can be used to describe the used three-dimensional unit cells, which can be seen in Figure 3. Structure a-3D is a regular cubic three-dimensional unit cell while b-3D and c-3D are eigenmodes of a-3D. As in 2D, structure b-3D can be described as the eigenmode with the chiral nodes where the sines’ turning points intersect at the nodal points because the nodal points do not possess a rotative reflection axis. Structure c-3D is named the eigenmode with the fixed nodal points where the extremal points of the sines meet in the nodal points. When oscillating, the nodal points of this eigenmode stay at their initial position without rotation or other movements.

### 2.3. Simulation Procedure

The unit cells were meshed with tetragonal mesh elements of a size between 0.18 and 1.0 for being able to resolve the different arcs with the same mesh settings. Material parameters of titanium (Young’s modulus E = 105 GPa, density *ρ* = 4940 kg m${}^{-3}$, Poisson’s ratio *ν* = 0.33) are used. Bloch-Floquet boundary conditions are applied pairwise to opposing surfaces. In 2D, the complete structure is additionally pinned in z-direction in order to disable movements along the z-axis. Eigenfrequency analysis is conducted along the boundaries of the 1st Brillouin zone (see Figure 1). In 2D, the first 50 dispersion branches were calculated with 25 points for each of the three directions. In 3D, the first 250 dispersion branches were calculated to account for the additional possible motions in z-direction. The path in $\vec{k}$-space for the three-dimensional cells can be seen in Figure 1b. For every point of a dispersion branch, the corresponding mode shape can be displayed.

For normalisation, the obtained eigenfrequencies $\omega_{i}$ are divided by the first pinned-pinned flexural resonance frequency $\omega_{0}$ of a beam with thickness *d* and length *l*, consisting of a material with elastic modulus *E* and density *ρ* to calculate the dimensionless frequency Ω:

(3) $$\Omega =\frac{\omega_{i}}{\omega_{0}}\;\;\;\;\;with\;\;\omega_{0}=\pi^{2}\sqrt{\frac{E\;d^{2}}{12\;\rho \;l^{4}}}$$

For better comparability between the results, the eigenfrequencies of structure b-2D, c-2D, b-3D and c-3D are also normalized with the $\omega_{0}$ belonging to the straight strut, although the length of their struts is slightly different.

## 3. Results

### 3.1. 2D Quadratic Unit Cells

**Figure 4:**
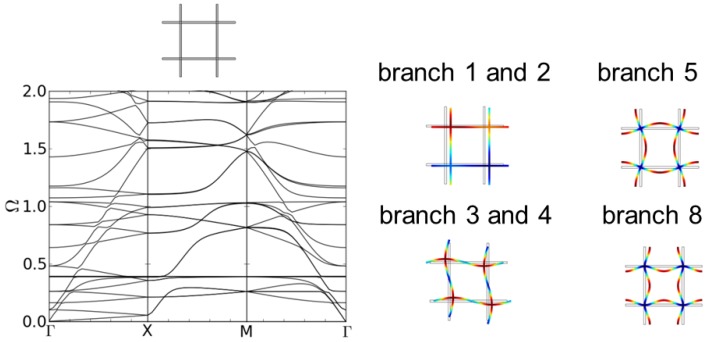
Dispersion relations of structure a-2D, a regular quadratic unit cell (l = 5.0 mm, d = 0.25 mm) and first eigenmodes at point Γ.

The dispersion results for the regular quadratic structure a-2D are shown in Figure 4. The normalized eigenfrequencies are plotted against the path Γ-X-M-Γ of the wave vector in $\vec{k}$-space (see Figure 1). No band gaps emerge. This is consistent with the results of Phani, *et al.* [9].

The corresponding eigenmode shapes for the first branches at point Γ in $\vec{k}$-space can be seen in Figure 4. While the first two eigenmodes correspond to a translation of the unit cell, for the third and fourth, the nodal points are moving and the struts begin to bend to follow the translation. The fifth eigenmode can be described as a bending of the struts due to a collective rotation of the nodal points that at the same time stay fixed at their initial position. The nodal points of the eighth eigenmode stay fixed at their initial position while the four struts between them bend alternatingly either all inwards or outwards.

**Figure 5:**
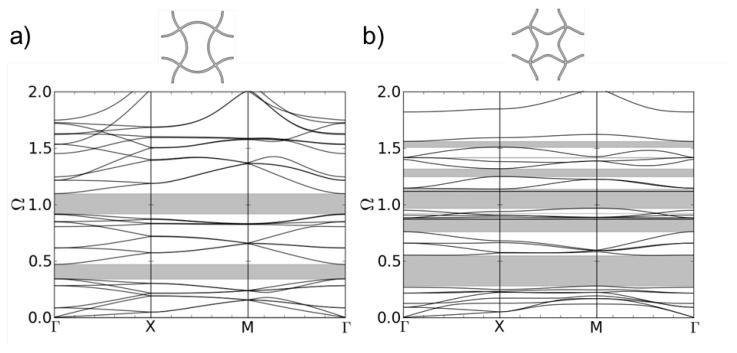
Dispersion relations of structure b-2D, the eigenmode with chiral nodal points (**a**); and structure c-2D, the eigenmode with fixed nodal points (l = 5.0 mm, A = 1.0 mm, d = 0.25 mm) (**b**).

**Figure 6:**
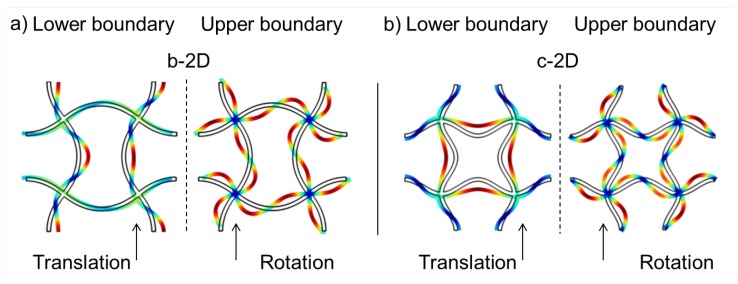
Eigenmodes at the lower and upper edge of the 1st band gap of structure b-2D (**a**) and c-3D (**b**).

The dispersion curves for the quadratically based structure b-2D can be seen in Figure 5a. In the considered frequency range, two band gaps develop. The first band gap emerges between the 8th and 9th dispersion branch. The corresponding eigenmodes are shown in Figure 6a. The eigenmode at the lower edge of the band gap can be described by an alternating translation of the two left or the two right nodal points, respectively. One pair of points moves towards each other and bends the connecting vertical strut further while the other pair of nodal points moves away from each other and unbends the connecting vertical strut between them to a plateau-like form. The two horizontal struts follow the motion of the two nodal points they connect.

The eigenmode at the upper edge of the band gap is a collective rotation of all nodal points in the same direction. The rotation direction changes between left and right while the nodal points stay fixed at their initial position all the time. The struts have to follow the rotation of the nodal points and bend. Körner and Liebold-Ribeiro showed in [12] that their bent shape is comparable to the first anti-symmetric mode of a single strut with hinged-hinged boundary condition.

The dispersion relation of the c-2D structure is shown in Figure 5b. Structure c-2D is the 8th eigenmode of structure a-2D (see Figure 4). The different clamping of the nodal points in structure b-2D and c-2D due to the different form of the bent struts leads to different eigenfrequencies of the eigenmodes depending on the direction of the incoming wave. This is reflected in the different form and density of the dispersion branches in Figure 5a,b. Now, eight band gaps of different sizes are showing up in the same range of frequencies as in Figure 5a. The first band gap occurs also between the 8th and 9th dispersion branch. While the lower frequency of the band gap is lower compared to structure b-2D, the size of the band gap is much larger. The corresponding mode shapes are depicted in Figure 6.

Again, the eigenmode at the lower limit of the band gap is a translation of nodes. All four nodal points move towards each other into the middle of the unit cell. The struts follow this movement by bending. Then, all nodal points move away from each other and stretch the sines in the struts so that they get unbent. As for structure b-2D, the eigenmode at the upper edge of the band gap is a collective rotation of the fixed nodal points to the left and then to the right.

### 3.2. 3D Quadratic Unit Cells

**Figure 7:**
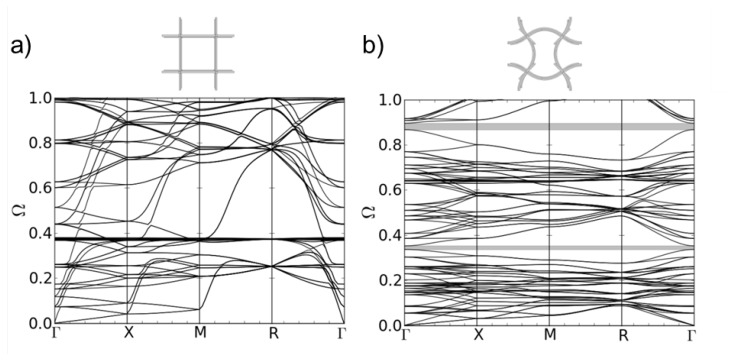
Dispersion relations of structure (**a**) a-3D (l = 5.0 mm, d = 0.25 mm); and (**b**) b-3D (l = 5.0 mm, A = 1.0 mm, d = 0.25 mm).

The dispersion relation for the a-3D quadratic three-dimensional unit cell is shown in Figure 7a. The eigenfrequencies are normalized with $\omega_{0}$ (see Equation 3) and plotted in dependancy of the position in $\vec{k}$-space on the path Γ-X-M-R-Γ (see Figure 1). No band gap is present. As movements in z-direction are now allowed, in the same range of frequencies, the number of dispersion branches strongly increases compared to the regular two-dimensional structure (see Figure 4).

**Figure 8:**
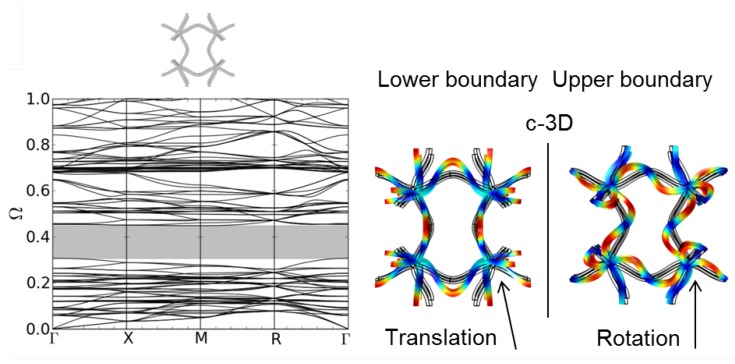
Dispersion relations of structure c-3D (l = 5.0 mm, A = 1.0 mm, d = 0.25 mm) and corresponding mode shapes at the lower and upper boundary of the first band gap.

For the b-3D structure, a small band gap emerges (see Figure 7b). Finally, the c-3D structure shows a pronounced gap at low frequencies (see Figure 8). The corresponding mode shapes at the lower and upper edge of the band gap can be seen in Figure 8. As in 2D, the mode shapes can be explained by a translation rotation of the nodal points, respectively. The different clamping of the nodal points in structure b-3D and c-3D due to the different form of the bent struts leads to different eigenfrequencies of the eigenmodes depending on the direction of the incoming wave. This is reflected in the different form and density of the dispersion branches in Figure 7b and Figure 8.

### 3.3. Variation of the 2D Unit Cell Dimensions

**Figure 9:**
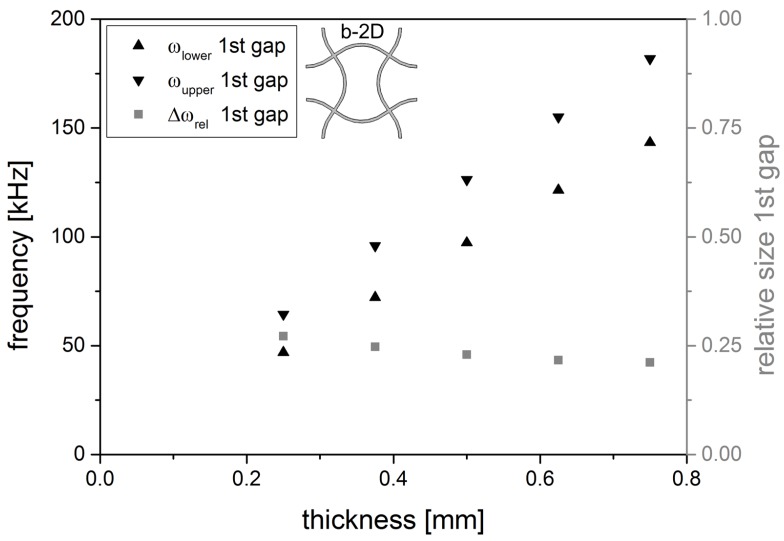
Lower and upper frequency of the 1st gap of structure b-2D as a function of the strut thickness (l = 5.0 mm, A = 1.0 mm) as well as the relative size $\Delta \;\omega_{rel}$ of the 1st band gap.

The band gap is also influenced by the dimensions of the unit cell. When changing the thickness of the struts of structure b-2D, the upper and lower frequency of the band gap is shifted to higher frequencies (see Figure 9). The thicker the strut, the stiffer the structure, the more difficult is the excitation of natural resonances. To get a measure for the size of the band gap, the relative size $\Delta \;\omega_{rel}$ of the band gap is calculated as

(4) $$\Delta \;\omega_{rel}\;=\;\frac{\omega_{upper}-\omega_{lower}}{\omega_{upper}}$$

with $\omega_{i}$ being the lower or upper limiting frequency of the band gap respectively.

**Figure 10:**
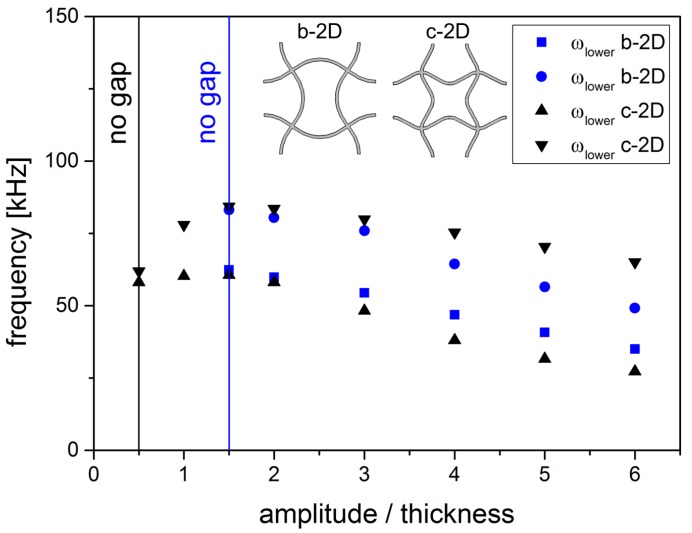
Upper and lower frequencies of the 1st band gap for structure b-2D and c-2D in dependancy of the amplitude A (l = 5.0 mm, d = 0.25 mm).

The dependancy on the second parameter of the structure, the amplitude of the sine, can be seen in Figure 10 where the upper and lower frequency of the band gaps for structure b-2D and c-2D for increasing ratios of amplitude to thickness are shown. For both structures, a certain amplitude is needed to open the band gap. In structure b-2D, the gap emerges at a relative amplitude larger than $\frac{3}{2}$. Once the band gap is opened, the limits of the band gap decrease and approach each other with increasing amplitude. In structure c-2D, the relative amplitude needs to be higher than $\frac{1}{2}$. In the beginning, the band gap is quite small but grows with increasing amplitude while the upper and lower limits of the band gap are shifted to higher frequency values at the same time. For relative amplitudes $\geq \frac{3}{2}$, the band gap width still increases, but the upper and lower limits of the band gap decrease. The frequency of the lower limit decreases faster than that of the upper limit. Thus, the width of the band gap grows with increasing amplitude A.

**Figure 11:**
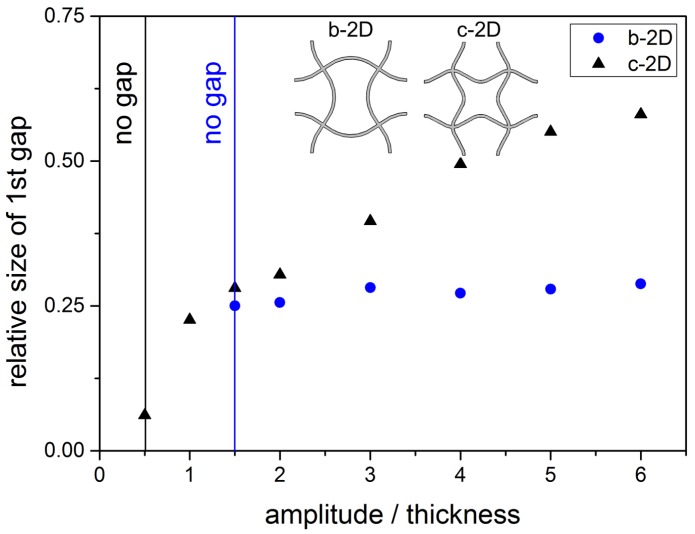
Relative size $\Delta \;\omega_{rel}$ of the 1st band gap for structure b-2D and c-2D in dependancy of the amplitude A of the bent struts (l = 5.0 mm, d = 0.25 mm).

This behaviour is illustrated in Figure 11 where the relative size of the band gap is plotted against the relative amplitude, the ratio of amplitude to thickness. The relative size is calculated according to Equation 4. For structure b-2D, once the band gap is open, the relative size stays approximately constant. For structure c-2D, two zones are visible: For A $<\frac{3}{2}$, a steep increase of the relative size of the band gap can be seen. For A $\geq \frac{3}{2}$, the growth of the relative size is less steep.

## 4. Discussion

### 4.1. Emergence of Band Gaps in Modified Quadratic Structures

**Figure 12:**
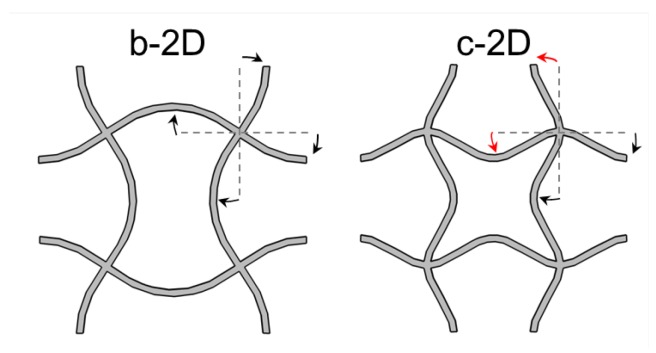
Bending directions of incoming struts for structure b-2D (**a**); and c-2D (**b**).

The movement of the cellular structure can be described by the movement of the nodal points. In principle, two different modes of movement, translation and rotation, are possible. Combinations of these two are also allowed. At low frequencies, wave propagation is based on node translation. The maximum frequency which can be realized in this way is determined by the strut length. Higher frequencies can only be realised by rotation of the nodal points. If the first collective rotation frequency of the nodal points is lower than the maximum translation frequency, no band gap develops. This is the case for the a-2D and a-3D structure. In the b and c structures rotation is hampered by the bent struts while translation is facilitated. That is, the frequencies for translation decrease while those for rotation increase whereby the gap opens. The reason for the opening of the band gap can thus be found in the shifting of the rotational eigenmode to a higher eigenfrequency. The b-structures already show gaps which are even more pronounced in the c-structures due to the clamping conditions at the node. At the nodes of b-structures, the bending direction of all incoming struts is identical (see Figure 12). Thus, only rotation in one direction is strongly hampered. In contrast, bending of the incoming struts varies in c-structures which leads to a restriction of rotation in all directions. As a consequence, the frequency of the collective rotation mode increases leading to a broad band gap.

Based on those findings, design principles for cellular structures with broad bandgaps can be deduced:

(a) the first eigenfrequency of the struts must be low: bent struts;
(b) the first collective rotational frequency must be high: clamping of the nodes.

### 4.2. Dependancy of Gap Boundaries on Strut Thickness

The linear increases in frequency at the upper and lower edge of the band gap (Figure 9) can be explained with Equation 3 that shows the linear dependancy of the first pinned-pinned flexural resonance frequency $\omega_{0}$ on the thickness d. The thicker the strut, the higher the $\omega_{0}$. The frequencies of the upper and lower edge of the 1st band gap of structure b-2D can be influenced by changing the strut thickness.

### 4.3. Dependancy of Gap Boundaries on Relative Amplitude

The amplitude of the struts determines the mechanical properties, especially the elastic modulus, of the struts. Thus, the excitation of the natural resonances is also affected. This can be used to influence the location and width of the band gap.

Band gaps in the b- and c-structures occur only if the ratio of amplitude to thickness is higher than a certain value (see Figure 10). This reflects that the effect of the hampered rotation of the nodal points needs a clamping of the nodal points that is strong enough. Otherwise, the structure is still too similar to a regular quadratic structure.

For structure b-2D, the decrease of translational and rotational frequency with increasing relative amplitude (see Figure 10) happens approximately with the same slope so that the relative size of the band gap stays approximately constant (see Figure 11. As all struts are bent in one direction, only one rotation direction is hindered. The rotational and translational eigenmodes are approximately eased by the same amount for increasing amplitudes.

For structure c-2D, an increase in the relative amplitude leads to an increase of both the translational and rotational frequency until a relative amplitude of $\frac{3}{2}$ is reached. This means that the translational and the rotational movements are more difficult to excite for increasing although low amplitudes. Hereby, rotation of nodes is even more difficult than translation as the slope of the rise in rotational frequency is steeper than that of translational frequency. Then, both frequencies decrease. Rotation is still hampered enough to open the band gap, but both translation and rotation is eased from a certain amplitude. The translational frequency decreases even faster than the rotational frequency. The translational movement of the nodal points towards each other gets easier faster than the rotation of the nodal points. This leads to the two domains of slope that can be seen in Figure 11. This observation can be explained by the two bending directions of the struts in structure c-2D. While the translation of the nodal points is facilitated with increasing amplitude, the rotation is less eased because the easement is distributed on the two rotation directions. The rotation to the left and to the right are both hindered in structure c-2D as two struts are bend in each direction.

## 5. Conclusions

Dispersion relations were calculated to study the dynamic behaviour of cellular materials that are based on regular quadratic and cubic unit cells. Eigenmode analysis can be used as a systematic way for obtaining variations of an underlying basic unit cell. It was shown how the replacement of straight struts by bent ones leads to the emergence of full phononic band gaps. The emergence of the first band gap can be traced back to a hindered rotation of the nodal points, induced by the bent struts. The hindering of single eigenmodes shifted their eigenfrequency to higher values and thus opened the band gap. The band gap location was modified by changing the unit cell parameters. The principle of designing the unit cell geometry in order to impede certain movements was deduced in 2D and successfully transferred to 3D. The influence of the unit cell parameters on three-dimensional quadratically based structures will be studied in future experiments.
